# Quantitative assessment of inner ear variation in elasmobranchs

**DOI:** 10.1038/s41598-023-39151-0

**Published:** 2023-07-24

**Authors:** Derek J. Sauer, Craig A. Radford, Christopher G. Mull, Kara E. Yopak

**Affiliations:** 1 Leigh Marine Laboratory, Institute of Marine Science, University of Auckland, Leigh, New Zealand; 2 Integrated Fisheries Laboratory, Department of Biology, Dalhousie University, Halifax, NS B3H 4R2 Canada; 3 Department of Biology and Marine Biology and the Center for Marine Science, University of North Carolina Wilmington, Wilmington, NC USA

**Keywords:** Evolutionary ecology, Inner ear

## Abstract

Considerable diversity has been documented in most sensory systems of elasmobranchs (sharks, rays, and skates); however, relatively little is known about morphological variation in the auditory system of these fishes. Using magnetic resonance imaging (MRI), the inner ear structures of 26 elasmobranchs were assessed in situ. The inner ear end organs (saccule, lagena, utricle, and macula neglecta), semi-circular canals (horizontal, anterior, and posterior), and endolymphatic duct were compared using phylogenetically-informed, multivariate analyses. Inner ear variation can be characterised by three primary axes that are influenced by diet and habitat, where piscivorous elasmobranchs have larger inner ears compared to non-piscivorous species, and reef-associated species have larger inner ears than oceanic species. Importantly, this variation may reflect differences in auditory specialisation that could be tied to the functional requirements and environmental soundscapes of different species.

## Introduction

Fishes possess the largest diversity in auditory anatomy among any vertebrate group (^1^; reviews by^2,3^). In both bony (Osteichthyes) and cartilaginous (Chondrichthyes) fishes, each inner ear is comprised of three semi-circular canals and three otoconial (or otolithic) end organs: the saccule, lagena, and utricle. While nearly all fishes share this generic inner ear blueprint, there is considerable variation between species in the size and shape of various structures, along with variation in the ultrastructure of the sensory epithelia^2–4^. The functional significance of this interspecific variation is unclear, although it is thought that morphological variation may reflect different hearing requirements, primary habitats, swimming movements, and life history traits associated with different species^5–8^. For example, some deep-sea fishes are thought to possess specialised inner ear morphology to cope with extreme environmental conditions, including relatively large semi-circular canals^9^ and saccules^10^, as well as laterally placed inner ears^11^.

The vast majority of what is known about variation in the inner ears of fishes is derived from bony fishes. Comparatively little is known about the inner ears of cartilaginous fishes (reviewed in^12^), especially in terms of interspecific variation^1,13–16^ and auditory capacity^17–21^. Many of the species in this group are difficult to study, due to their large size, high mobility, and difficulty of being maintained in captivity^22^, which has led to a lag in our understanding of their auditory anatomy compared to other fishes. However, cartilaginous fishes represent some of the first vertebrates to evolve the capacity for sound detection and can provide key insights into the early evolution of the auditory system in vertebrates.

Elasmobranchs (sharks, rays, and skates) comprise the majority of cartilaginous fishes and are thought to possess the same generic inner ear structures as bony fishes; however, the elasmobranch inner ear contains a fourth end organ, the macula neglecta, which lacks an otoconial mass^23,24^. The macula neglecta is absent or diminutive in many bony fishes and its unique prominence in elasmobranchs has led to the belief that it holds a significant role in auditory function in this group^25^. There is evidence of remarkable vibrational sensitivity in the macula neglecta of some elasmobranchs^26–28^, although its precise functional role within the auditory system of this group remains unclear.

Inner ear anatomy has been described in several elasmobranch species (*see *^12^* for review*); however, relatively little is known about the degree to which inner ear morphology varies between species and how this variation relates to phylogenetic position and/or ecological parameters. In fact, there have only been two studies that quantitatively describe interspecific variation in inner ear soft tissues in elasmobranchs. Corwin^13^ first examined the inner ears of six elasmobranchs species, including four sharks (Selachii) and two rays (Batoidea), and suggested that interspecific variation does not reflect phylogenetic relationships, but rather is linked to ecological differences between species. Specifically, Corwin^13^ proposed that species which feed by “raptorial predation” have a larger, dorsally positioned macula neglecta that is seemingly more specialised for hearing compared to species that feed predominantly on invertebrates on the seafloor. Building upon Corwin’s^13^ work, Evangelista et al.^14^ later examined an additional 17 elasmobranch species (9 rays and 8 sharks) and proposed four inner ear morphological categories, and suggested that variation in the size of different inner ear structures was likely the result of a combination of phylogenetic and functional factors (e.g., feeding strategy, behaviour)^14^. Together, these studies have established broad diversity in elasmobranch inner ear morphology; however the predictions from these studies have yet to be tested using a comparative framework, and the auditory anatomy of the majority of the *c.* 1202 species of elasmobranchs^29^ have yet to be examined. Thus, the extent to which inner ear morphology varies among elasmobranchs remains largely unknown, as do the factors that potentially explain the variation among species.

Elasmobranchs are ancestral predators that inhabit a wide range of ecological niches, in both freshwater and marine habitats. They have adapted to complex environmental conditions across diverse habitats^30^ and possess varying body forms and lifestyles. Correspondingly, there is considerable morphological variation in all elasmobranch sensory systems examined thus far, including the visual, olfactory, and electrosensory systems^31–35^, as well as the brain regions receiving primary afferents from these sensory systems^36–39^. Variation across these systems has been correlated with a variety of ecological factors, including primary habitat, lifestyle, and diet^40–43^, and has been used to make inferences about the relative importance of different sensory systems within and across species. Morphological variation in inner ear morphology may similarly reflect ecological and/or behavioural differences between species and provide insight into auditory specialisation in elasmobranchs, although empirical evidence is lacking. Therefore, the aim of the current study was to use phylogenetically-informed multivariate analyses to characterise interspecific variation in the inner ear of elasmobranchs and assess potential ecological drivers of morphological variation.

## Results

### pGLS scaling

**Figure 1:**
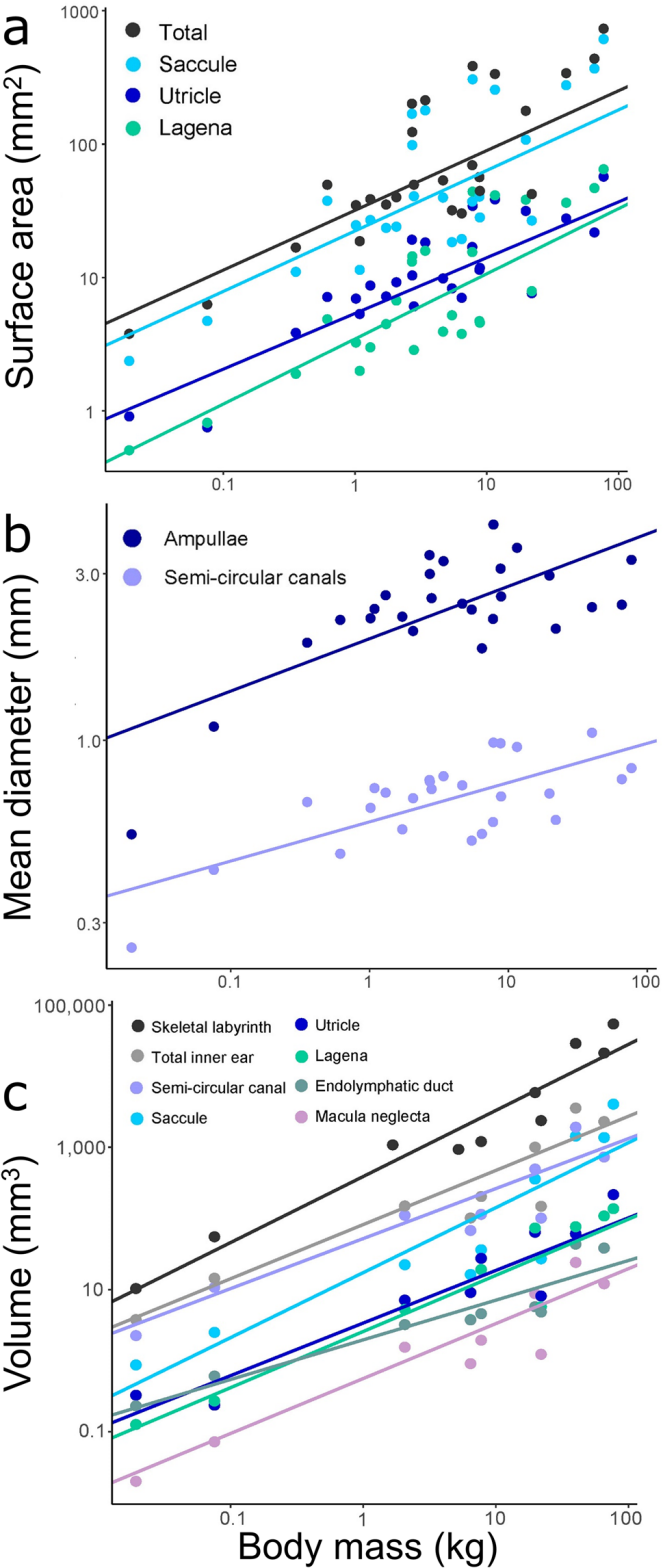
Interspecific scaling relationships of body size and inner ear measurements. Scaling relationships (logarithmic axes) from pGLS models between (**a**) body mass and surface area of the saccule, lagena, and utricle (and their combined total surface area) across 26 elasmobranch species. Total surface area = 0.45x + 0.16. Saccule = 0.45x – 0.01. Lagena = 0.49x – 0.93. Utricle = 0.42x – 0.52; (**b**) body mass and mean diameter of the semi-circular canals and ampullae from 26 elasmobranch species. Mean ampulla diameter = 0.15x – 0.16. Mean semi-circular canal diameter = 0.11x – 0.57; (**c**) and body mass and volume of different inner ear structures in 10 species. Saccule = 0.91x – 1.50. Lagena = 0.79x – 1.95. Utricle = 0.74x – 1.69. Macula neglecta = 0.77x – 2.57. Endolymphatic duct = 0.56x – 1.38. Horizontal canals = 0.70x – 0.39. Soft labyrinth = 0.76x – 0.37. Skeletal labyrinth = 0.93x – 0.20. For full regression outputs, see Tables S1 and S2.

All linear and area inner ear measurements exhibited a significant, positive relationship with body mass across 26 species (Fig. 1a,b, Table S1) and scaled with negative allometry (i.e., slope < 0.66 for surface areas, slope < 0.33 for linear measurements), whereby inner ear components increased with body size, but at a slower rate. Similarly, the volume of all inner ear components also increased with body mass across 10 species (Fig. 1c, Table S2) and scaled with negative allometry (slope < 1).

### Phylogenetic comparisons

**Figure 2:**
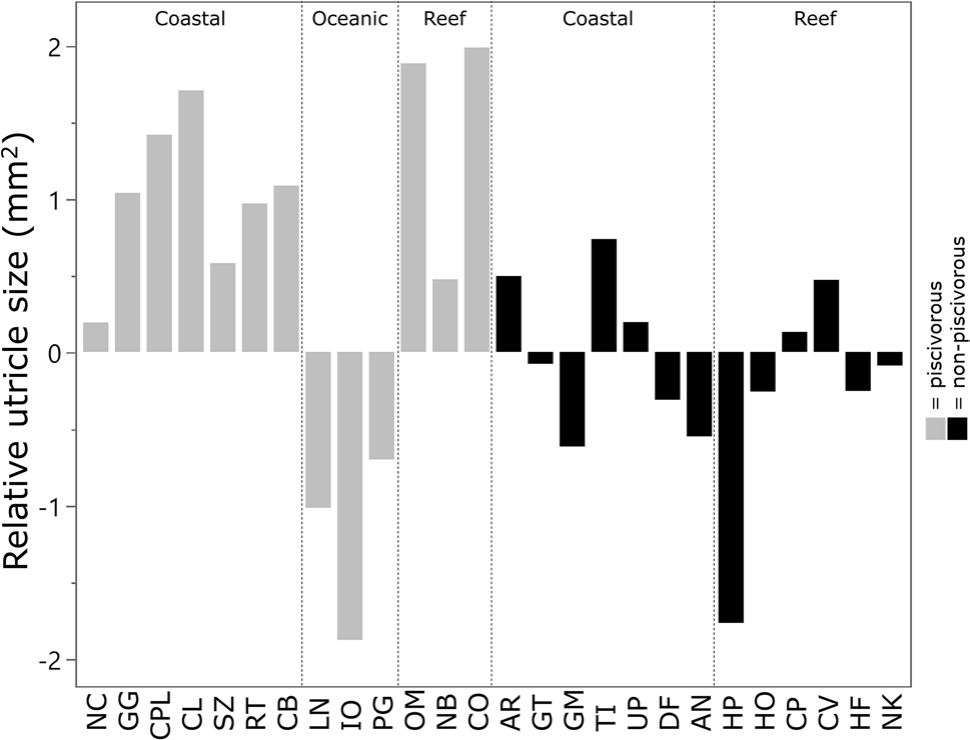
Utricle size in elasmobranchs. Relative utricle size (residuals from pGLS models against body mass) for each species, grouped by diet (coloured) and habitat (segmented). For species abbreviations, see Table 3.

**Figure 3:**
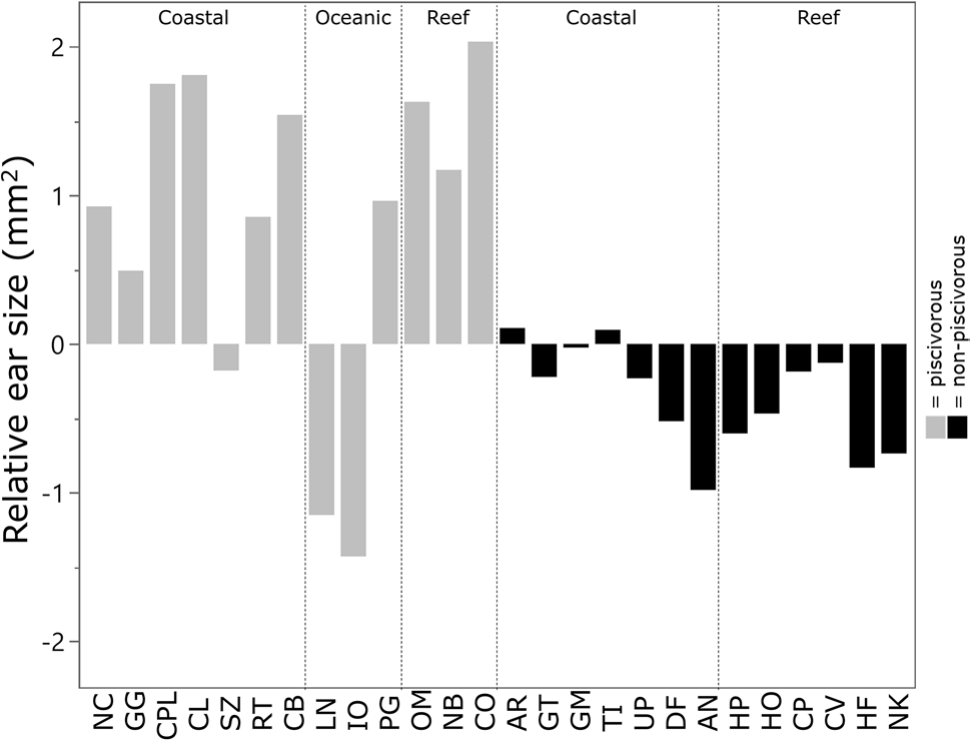
Ear size in elasmobranchs. Relative ear size (residuals from pGLS models against body mass) for each species, grouped by diet (coloured) and habitat (segmented). For species abbreviations, see Table 3.

There were minimal significant differences in the relative size of inner ear components (based on phylogenetically-corrected residuals) between habitat, lifestyle, and diet categories (Tables S4, S5). However, significant differences were detected in the surface area (F_2,23_ = 4.54 p = 0.043) and volume of the utricle between habitats (F_2,7_ = 6.69, p = 0.039), with oceanic species having smaller utricles compared to coastal species (p = 0.06 and p = 0.03, respectively) (Fig. 2). The volume of the semi-circular canals also differed between lifestyles (F_2,6_ = 6.77, p = 0.029), with pelagic species having significantly smaller canal volumes than benthopelagic species (p = 0.039). In addition, piscivores trended towards larger ear size residuals than non-piscivores (F_1,24_ = 13.07, p = 0.133), where oceanic species with relatively small inner ears represented piscivorous outliers and likely masked biologically relevant differences (Fig. 3).

There were also minimal significant differences in eigenvalues from pPCA between habitat, lifestyle, and diet categories (Table S6); however, PC1 eigenvalues were significantly smaller (note: negative values indicate larger ears) in piscivorous species compared to non-piscivorous species (F_1,24_ = 25.48, p = 0.047).

Total ear size (λ = 1.06, p < 0.001), as well as surface area of the saccule (λ = 1.09, p < 0.001), lagena (λ = 0.95, p < 0.001), and utricle (λ = 0.61, p = 0.044) were significantly influenced by phylogenetic relatedness. However, the mean diameter of the semi-circular canals (λ = 0.03, p = 0.922) and mean ampulla diameter (λ = 0.00, p = 1.00) were not significantly influenced by phylogenetic relatedness.

### Phylogenetic PCA

#### PC1—Inner ear size

**Figure 4:**
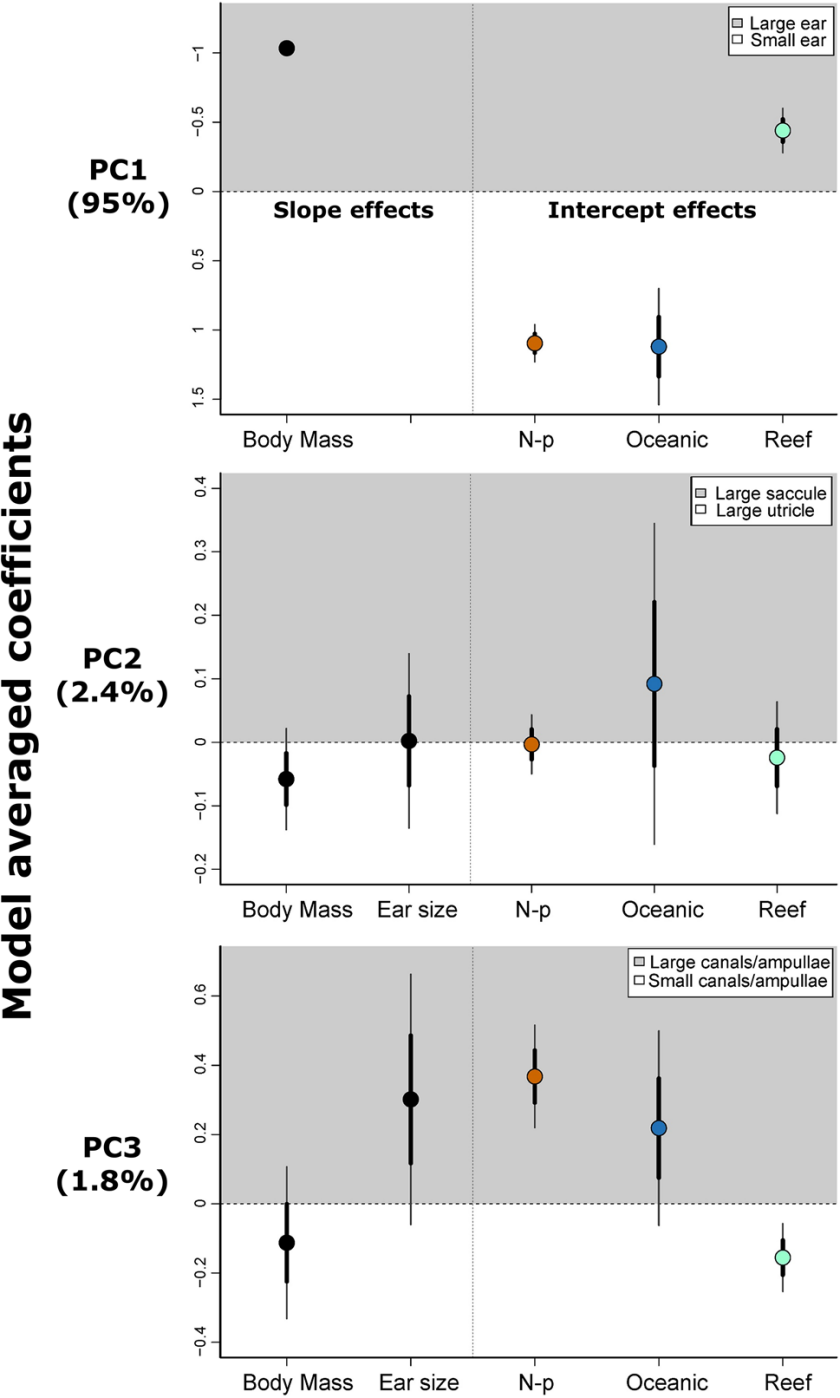
Model-averaged coefficients for main principal components. The slope effects from body size and total ear size, as well as the intercept effects from diet and primary habitat (n = 26 species). Intercept effects are relative to a reference level of coastal, piscivorous species. Note that total ear size was not included as a predictor for PC1, as this principal component characterises variation in total ear size. N-p = non-piscivorous.

Inner ear structure varied across three main axes (principal components) in 26 species of elasmobranchs. The first axis was related primarily to total ear size, seen by large negative loadings for total ear size and all ear structures (Table 1A), and explained the majority (95%) of the variance. Body mass had a large allometric effect on total ear size (model averaged coefficient ± standard error, − 1.03 ± 0.03) (Fig. 4). Piscivorous species had significantly larger relative ear sizes compared to non-piscivores (t = − 16.13, p < 0.001), while oceanic species had significantly smaller relative ear sizes compared to both coastal and reef-associated species (Table 2). In addition, reef-associated species had significantly larger relative inner ears than coastal species (Table 2).

**Table 1:** Eigenvalues for inner ear structures.

Structure	PC loadings		
	PC1	PC2	PC3
A. Linear measurements (n = 26)			
Total ear size	− 0.995	0.090	− 0.013
Saccule	− 0.983	0.185	− 0.010
Lagena	− 0.972	− 0.170	− 0.144
Utricle	− 0.960	− 0.214	0.143
Mean canal diameter	− 0.845	0.108	0.327
Mean ampulla diameter	− 0.824	− 0.034	0.491
Standard deviation	0.062	0.010	0.008
Proportion variance	0.947	0.027	0.017
Cumulative variation	0.947	0.974	0.991
Lambda (λ)	0.455	–	–
B. Volume measurements (n = 10)			
Total ear volume	− 0.997	0.084	0.024
Saccule	− 0.983	0.159	− 0.088
Lagena	− 0.991	− 0.087	− 0.075
Utricle	− 0.960	− 0.275	− 0.015
Macula neglecta	− 0.996	− 0.016	0.082
Semi− circular canals	− 0.991	0.065	0.120
Standard deviation	0.152	0.021	0.012
Proportion variance	0.974	0.019	0.006
Cumulative variation	0.974	0.993	0.999
Lambda (λ)	0	–	–

Eigenvalues for the three main principal components, using (A) linear measurements from 26 species and (B) volumetric measurements from 10 species.

**Table 2:** Habitat effects.

	Coastal	Oceanic	Reef
PC1			
Coastal	–	− 5.25**	5.42**
Oceanic	**− 1.12**	–	9.11**
Reef	**0.44**	**1.56**	–
PC2			
Coastal	–	− 0.715	0.54
Oceanic	− 0.092	–	0.875
Reef	0.024	0.116	–
PC3			
Coastal	–	− 1.527	3.121*
Oceanic	− 0.219	–	2.535
Reef	**0.155**	0.374	–

Post hoc testing (pairwise comparisons) of estimated marginal means for each principal component across habitat categories. Values above the diagonal display test ratios, while values below the diagonal are contrast estimates. Significant values are in bold (** denotes p < 0.001, * denotes p < 0.05).

#### PC2—Utricle vs. saccule

The utricle versus saccule axis reflected independent variation between surface area of the utricle (− 0.21) and the saccule (0.18), and explained half of the remaining variance (2.4%; 97% cumulative) (Table 1A). There was moderate support for a negative allometric effect of body size (− 0.06 ± 0.04), and weak support of an intercept effect for oceanic species (0.09 ± 0.13) (Fig. 4), likely a result of the wide confidence interval derived from n = 3 oceanic species. There were no significant intercept effects of diet (t = − 0.17, p = 0.87) or habitat (Table 2).

#### PC3—Mean semi-circular canal and ampulla diameter

The semi-circular canal axis reflected differences in the mean diameters of the semi-circular canals and their ampullae relative to other components of the inner ear (Table 1A), and explained 1.8% of the variance (99% cumulative). There was moderate support for a positive allometric effect of ear size on canal and ampulla mean diameters (0.30 ± 0.18) (Fig. 4). Non-piscivorous species had significantly larger semi-circular canal and ampulla diameters compared to piscivores (t = − 4.88, p < 0.001). Reef-associated species had significantly smaller semi-circular canal and ampulla diameters compared to coastal species (t = 3.12, p = 0.015), although this was largely driven by relatively large canal and ampulla diameters in coastal, non-piscivorous species.

### Volume measurements

Phylogenetic PCA of volumetric measurements from the smaller dataset of 10 species supported the findings from linear measurements, with three axes of variation that closely resembled those from linear measurements (Table 1B). The first axis was related to total ear volume, the second axis represented the negative relationship between utricle and saccule volume, and the third axis was related to the volume of semi-circular canals.

## Discussion

Quantitative assessment of morphological variation in the inner ears of elasmobranchs is rare, leaving a gap in our understanding of the auditory system of fishes and the early evolution of auditory function in gnathostomes. Here, interspecific variation in the inner ear morphology of 26 elasmobranch species was characterised using multivariate comparative analyses. Results indicate that inner ear morphology in elasmobranchs varies across three main axes. The majority of variation is attributable to body size, as all inner ear structures exhibited positive, hypoallometric scaling with body size. The remaining variation reflects varying allometries between the saccule and the utricle, and the relative size of semi-circular canals and their associated ampullae. Importantly, these axes of variation are influenced by diet and primary habitat, establishing that, similar to other sensory systems (and the brain) in elasmobranchs^40–43^, morphological variation in the auditory system is influenced by ecological traits associated with different species.

### Bioimaging

Magnetic resonance imaging (MRI) is a powerful, non-invasive method for obtaining high-resolution 3D morphological data, and it has been widely applied to studying various systems in fishes^44,45^, including those in elasmobranchs^46–50^. Extracting the inner ears of fishes is tedious and time consuming, and often results in destruction of surrounding tissues (e.g., eyes, brain), if not the inner ears themselves. MRI allows for complete non-destructive in situ assessment of the sample, direct measurement of individual inner ear structures, and the acquisition of data that can readily be used for quantitative, comparative analyses.

To our knowledge, this study is the first to use MRI to obtain in situ characterisations of the soft tissues of the fish inner ear, an advancement which can greatly expand our understanding of fish auditory systems. Prior studies have used measurements of the skeletal labyrinth (via computed tomography, CT) to examine inner ear morphology in elasmobranchs^51–57^. However, the present study indicates that soft tissue fills only a small proportion of the skeletal labyrinth, and that there is considerable interspecific variation in the ratio of soft to skeletal volume (see Table S8). While the high costs associated with using MRI can be prohibitive, the use of this method should be considered in future studies on the inner ears of elasmobranchs and other fishes, especially as MRI technology continues to develop and become more financially accessible.

### Inner ear organisation

The size of inner ear structures generally scale hypoallometrically (i.e., with negative allometry) in relation to body size in vertebrates, whereby slopes of linear measurements with respect to body mass range from 0.05 to 0.24 throughout vertebrates^58–60^. In elasmobranchs, a positive correlation between the size of inner ear maculae and body size is relatively well-established within individual elasmobranch species throughout ontogeny^24,61–66^; however, no studies have explored how growth of inner ear structures relates to overall body size across a wide range of species. In this study, inner ear structures scale hypoallometrically with respect to body mass in elasmobranchs, such that inner ear size increases with body mass across species, but at a slower rate (slope =  ~ 0.13 for linear measurements). This is in line with expectations, as it agrees with scaling of inner ear structures in other vertebrates, and we similarly see hypoallometric scaling of other peripheral sensory structures in elasmobranchs, such as eye size^32^ and olfactory rosette and sensory epithelium size^33,67^. Further, these patterns are maintained centrally as well, whereby the brain scales hypoallometrically with the body and many brain regions in elasmobranchs (and other vertebrates) similarly show a negative scaling relationship with brain mass^68,69^. The findings of this study confirm similar allometric growth in the peripheral auditory system of elasmobranchs.

Across vertebrates, the semi-circular canals of the inner ear are responsible for transducing and processing information related to body position and motion in three-dimensional space^70,71^. They provide an animal with information about how it moves within its environment and facilitate coordination of posture, body movements, and eye stabilisation during movement^71^. The diameter of the each canal duct is thought to positively scale with the radius of curvature^59^, while both canal diameter and radius of curvature correlate positively with sensitivity across vertebrates^59,72,73^. In mammals and birds, the size of the semi-circular canals (i.e., canal diameter and radius of curvature) is also positively correlated with locomotor behaviour, with more agile species having larger semi-circular canals^71,74,75^. However, such a relationship has not been established in fishes, which have larger semi-circular canals relative to their body size than other vertebrate groups^59^, and possess a comparatively restricted ability to make swift head movements^76^. Additionally, the utricle is thought to serve alongside the semi-circular canals in mediating body equilibrium in fishes^70,77^, as numerous experimental studies have documented the importance of the utricle to vestibular function^76,78–80^.

The functional roles of the different structures of the elasmobranch inner ear are largely unknown and there is likely substantial overlap of vestibular and auditory function within each of the inner ear end organs^4,5,77^. However, in fishes, it is generally accepted that the ‘upper’ ear (consisting of the semi-circular canals and the utricle) contributes largely to the vestibular system, while the ‘lower’ ear (consisting of the saccule, lagena, and macula neglecta in elasmobranchs) serves significantly in auditory tasks^2,5,70,81^. While there are disparities to this pattern in diverse fishes^82–84^, there is an array of support for this anatomical and functional segregation in the inner ear^77,85^, including physiological evidence from elasmobranchs suggesting the saccule and macula neglecta serve primary roles in audition^28^. In addition, pressure transduction mechanisms (i.e., otophysic connections) in teleosts are usually associated with the saccule, further suggesting an auditory role in the saccule of fishes^77^.

In fishes, larger inner ear structures may be indicative of increased functional capacity, as larger sensory epithelia contain more sensory receptors^61,63,65,66,86,87^. Indeed, the number of sensory receptors (i.e., hair cells) is positively correlated with sensitivity in fishes^61,88–91^ and the size of the otoconial organs has been directly tied to acoustic functionality in some species^79,92,93^. This trend may apply to other sensory systems of fishes, as the size of peripheral sensory organs often scales positively with the brain region that receives associated primary afferents from the sensory receptors, and both can serve as an indicator of the relative importance of a particular sensory system within species^39,94–96^. In fact, larger relative size of sensory organs is generally suggestive of specialised function throughout vertebrates^97–101^. Accordingly, relatively large inner ears in some elasmobranchs may confer the functional significance of auditory signals and/or improved auditory capacity, as compared to species with relatively small inner ears.

Corwin^13^ observed larger macula neglectas in elasmobranch species that capture free-swimming prey, and hypothesised that selection for increased auditory sensitivity in piscivorous species could drive this morphological trend. In the current study, there were no differences in the relative volume of the macula neglecta related to piscivory, and the volume of the macula neglecta did not explain a significant amount of variation in the inner ear (although these analyses were limited to data from 10 species). Piscivorous species did, however, possess relatively large total ear size compared to non-piscivorous species. This finding may align with Corwin’s hypothesis, as this difference in ear size could be the result of morphological adaption for the detection and utilisation of specific acoustic signals. Piscivorous sharks are attracted to certain low-frequency sounds^102–105^, thought to resemble those of a struggling (e.g., wounded or sick) fish, and behavioural evidence from lemon sharks (*Negaprion brevirostris*) indicates that acoustic signals aid these sharks in locating prey^106,107^. Whether piscivorous species possess enhanced or specialised auditory capabilities compared to non-piscivorous species remains to be determined, but the morphological distinctions observed in this study suggest the relative importance of audition in piscivorous species may be higher than species that predate primarily on the seafloor.

Connections between locomotor behaviour and semi-circular canal dimensions are widespread in vertebrates. In mammals, the size of the semi-circular canals is correlated with afferent sensitivity^72^. Further, mammals^71^, birds^75^, and salamanders^108^ show correlations between semi-circular canal dimensions and locomotor agility. In fishes, however, links between semi-circular canal dimensions and locomotor behaviour are lacking. In fact, pike (*Esox *sp.) exhibit no correlation between sensitivity of the semi-circular canals and canal size^109^. The semi-circular canals of fishes are also relatively large compared to other vertebrates groups^59,109^, suggesting that fishes may be distinct from other vertebrates in canal morphology. In the current study, the inner ears of piscivorous species were characterised by relatively small semi-circular canals and ampullae compared to non-piscivores. This contradicts evidence from other vertebrate groups, which predicts piscivores that feed off the substrate should possess relatively large semi-circular canal diameters to facilitate the vestibular requirements associated with catching agile prey in an open water environment, similar to birds that perform highly agile aerial manoeuvres^75^. Alternatively, the structurally homogenous open-water environments that many piscivorous sharks feed in (excluding reef-associated species) may reduce the need for more nuanced vestibular cues compared to fishes that must manoeuvre their bodies throughout more complex habitats (e.g., obstacles, shelters, crevices)^76^. Widespread evidence for this hypothesis is lacking, although salamander species that occupy structurally complex habitats are known to possess relatively large semi-circular canals^108^, as do some deep-sea fishes that perform tilted hovering manoeuvres^9^. When also considering the utricle and its potential vestibular capacity, the results here do suggest some support for this hypothesis; of the 13 piscivorous species in this study, the true oceanic species, such as *Isurus oxyrinchus* and *Prionace glauca*, possess relatively small utricles, while reef-associated species (e.g., *Carcharhinus obscurus* and *Orectolobus maculatus*) that inhabit structurally complex environments have relatively large utricles. However, findings in the semi-circular canals are contradictive, as reef-associated species possess relatively small semi-circular canals and ampullae compared to oceanic species. Interpreting the results from the utricle and semi-circular canals collectively lends confusion, but highlights that there may be trade-offs in various structures of the inner ear, and that much more work is required to determine which aspects may confer functional significance.

In addition to the relationship between inner ear structure and piscivory in elasmobranchs, variation in inner ear morphology is also partially explained by primary habitat. Connections between habitat and morphology have been established in other sensory systems of elasmobranchs^41–43^, and suggest variation in the relative importance of these systems across different ecological niches. For example, oceanic elasmobranchs typically possess relatively large eyes^32^ and correspondingly large regions of the brain (optic tecta) that receive the majority of primary afferents from the retinal ganglion cells^38^, while benthopelagic elasmobranchs tend to possess relatively large olfactory sensory surface areas^33^. In the auditory system, however, there is a paucity of data available on inner ear anatomy in elasmobranchs with which to test these predictions in a comparative context^14^. Historically, it has been thought that the primary driver of inner ear morphology in fishes was related to vocalisations between conspecifics, as these were the only sound signals of known biological significance^110^. However, recent experimental data have revealed that environmental soundscapes, which represent all the sound characteristics of an environment, provide information to fishes that can influence their behaviour^111–113^. As such, soundscapes may influence the adaptation of the inner ear and auditory capabilities in fishes throughout evolution^110^.

The correlations between inner ear morphology and habitat may be indicative of the role that environmental soundscapes play in shaping auditory structures in elasmobranchs. For example, the relatively larger inner ears observed in reef-associated species (compared to both coastal and oceanic species) (Fig. 3) could be a morphological adaptation to the relatively high levels of ambient sound present in reef habitats, particularly given this enlargement is likely not due to the size of the semi-circular canals, which are reduced in this group. Temperate and coral reefs represent relatively loud marine habitats^114–116^, with ambient sound from both abiotic and biotic sources covering a wide range of frequencies (^117^ and references therein). Many of the sound frequencies common to coral reefs overlap with the known hearing range of elasmobranchs^17,18,118,119^ and travel relatively large distances^120^. These sound sources may contain information that is advantageous for predator avoidance, mating, prey capture, migration, and recruitment in fishes, and there is evidence of bony fishes using reef sound to inform their behaviour^111–113^. Thus, the relatively large ears of reef-associated elasmobranchs may be an adaptation of the auditory system to detect and utilise specific sound information from reef habitats. Such a connection between sensory system morphology and environmental soundscapes is a predicted outcome of the sensory drive hypothesis^121^, which postulates that sensory systems co-evolve with relevant signals.

The soundscapes of open-ocean environments are distinct from coastal and reef environments^122,123^. Importantly, abiotic sound sources (in the 50–500 Hz range) are 5 to 15 decibels louder in coastal habitats compared to the open ocean^124^. Low levels of ambient sound in oceanic soundscapes may partially explain the relatively small inner ears in oceanic elasmobranchs compared to both coastal and reef species (Figs. 3, 4). It could also be that conditions in oceanic habitats are favourable for utilising other sensory cues, and oceanic species depend on sound to a lesser extent. Oceanic elasmobranchs are thought to rely heavily on vision for prey capture, as they are commonly found in the uppermost, clearest portion of the ocean (photic zone), while many coastal fishes rely less on vision and more on other sensory systems^94^. Epipelagic species also possess relatively large eyes and brain regions associated with visual input^32,38^. As such, the relatively small ears in some oceanic elasmobranchs may reflect the specialisation of other, non-auditory senses. Pelagic teleosts have relatively small saccular otoliths, which has been proposed to reflect reduced auditory capabilities compared to bottom-dwelling species^76,125,126^. However, this is largely speculative, as relatively little is known about the hearing abilities of oceanic elasmobranchs^17^, and one of the oceanic species in this study, *P. glauca*, actually possessed a relatively large inner ear. Clearly an expanded dataset that includes a wide range of taxonomically diverse, oceanic species is required to determine whether consistent patterns exist in inner ear morphology in pelagic sharks.

The extent of morphological variation in the auditory system of elasmobranchs, along with its functional and ecological implications, is largely unknown. This study, which presents one of the first quantitative comparisons of inner ear morphology in elasmobranchs, has demonstrated that variation in the auditory system across 26 elasmobranch species can be characterised by three primary axes that are influenced by the diet and habitat of different species. Piscivorous elasmobranchs have relatively larger inner ears compared to non-piscivorous species, which may indicate greater reliance on the auditory system for capturing mobile prey. Reef-associated species have significantly larger inner ears than oceanic species, potentially revealing an influence of soundscapes and/or the importance of the auditory cues in spatially complex habitats. Overall, the morphological patterns in this study suggest a connection between the selection pressures associated with primary habitats and the morphology of the elasmobranch auditory system. While experimental data related to the soundscapes of different habitats, the hearing abilities of a range of species, and the vestibular requirements associated with environmental complexity are needed to formally assess the functional significance of this morphological variation, the findings here reveal ecological factors that partially explain variation in the inner ears of elasmobranchs.

## Methods

### Data collection

Body size information and inner ear measurements were collected from 10 elasmobranch species (Table 3). These animals were obtained opportunistically from local commercial fishers in Leigh, New Zealand. In instances where body mass could not be measured, published length–weight relationships^127^ were used to estimate mass. The inner ears (within the otic capsules) of each specimen were preserved by immersion fixation in 4% paraformaldehyde (*following 65*, *66*). The data from these 10 newly examined species were then combined with data from 16 additional species collated from Evangelista et al.^14^, for a total of 26 species (Table 3). The experimental protocols in this study were approved by the University of Auckland’s Animal Ethics Committee (protocol #002066). All methods were performed in accordance with the relevant guidelines and regulations of the University of Auckland. Reporting in this manuscript follows the recommendations in the ARRIVE guidelines.

**Table 3:** Species information.

Species	Common name	Total length (cm)	Mass (kg)	n	Lifestyle	Habitat	Diet	Source
*Aetobatus narinari (AN)*	White spotted eagle ray	70.8 ± 15.7	5.4 ± 3.3	2	Benthopelagic	Coastal	Non-piscivorous	Evangelista et al.^14^
*Aptychotrema rostrata (AR)*	Eastern shovelnose ray	72.5 ± 2.1	1.0 ± 0.1	4	Benthic	Coastal	Non-piscivorous	Evangelista et al.^14^
*Carcharhinus brachyurus (CB)*	Bronze whaler	201.7 ± 19.5	77.0 ± 19.4	3	Pelagic	Coastal	Piscivorous	This study
*Carcharhinus leucas (CL)*	Bull shark	85.5	2.7	1	Benthopelagic	Coastal	Piscivorous	Evangelista et al.^14^
*Carcharhinus obscurus (CO)*	Dusky shark	89.6	7.8	1	Benthopelagic	Reef	Piscivorous	Evangelista et al.^14^
*Carcharhinus plumbeus (CPL)*	Sandbar shark	77.0 ± 1.0	3.4 ± 1.0	3	Benthopelagic	Coastal	Piscivorous	Evangelista et al.^14^
*Cephaloscyllium isabellum (CI)*	New Zealand carpet shark	73.1 ± 0.6	2.1 ± 0.0	3	Benthic	Reef	Non-piscivorous	This study
*Chiloscyllium punctatum (CP)*	Brown banded bamboo shark	82.6 ± 6.5	1.7 ± 0.3	3	Benthic	Reef	Non-piscivorous	Evangelista et al.^14^
*Dasyatis fluviorum (DF)*	Estuary stingray	57.0 ± 10.2	8.8 ± 5.2	5	Benthopelagic	Coastal	Non-piscivorous	Evangelista et al.^14^
*Galeorhinus galeus (GG)*	School shark	165.0 ± 2.9	19.7 ± 1.0	3	Benthopelagic	Coastal	Piscivorous	This study
*Glaucostegus typus (GT)*	Giant shovelnose ray	112.3 ± 7.1	4.7 ± 0.8	3	Benthic	Coastal	Non-piscivorous	Evangelista et al.^14^
*Gymnura micrura (GM)*	Smooth butterfly ray	48.5 ± 32.5	2.8 ± 2.4	2	Benthic	Coastal	Non-piscivorous	Evangelista et al.^14^
*Hemiscyllium ocellatum (HO)*	Epaulette shark	18.9 ± 0.8	0.02 ± 0.0	3	Benthic	Reef	Non-piscivorous	This study
*Heterodontus portusjacksoni (HP)*	Port Jackson shark	25.7 ± 0.9	0.08 ± 0.0	3	Benthic	Coastal	Non-piscivorous	This study
*Himantura fai (HF)*	Pink whipray	70.0 ± 2.0	8.9 ± 0.3	2	Benthopelagic	Reef	Non-piscivorous	Evangelista et al.^14^
*Isurus oxyrinchus (IO)*	Short fin mako	153 ± 13.5	21.9 ± 5.3	3	Pelagic	Oceanic	Piscivorous	This study
*Lamna nasus (LN)*	Porbeagle	85.4	6.4	1	Pelagic	Oceanic	Piscivorous	This study
*Negaprion brevirostris (NB)*	Lemon shark	81.0 ± 1.0	2.7 ± 0.0	2	Benthopelagic	Reef	Piscivorous	Evangelista et al.^14^
*Neotrygon kuhlii (NK)*	Blue spotted maskray	30.9 ± 3.3	1.1 ± 0.3	3	Benthopelagic	Reef	Non-piscivorous	Evangelista et al.^14^
*Notorynchus cepedianus (NC)*	Seven gill shark	228.0	40.0	1	Benthopelagic	Coastal	Piscivorous	This study
*Orectolobus maculatus (OM)*	Spotted wobbegong	123.5 ± 0.7	11.5 ± 0.3	5	Benthic	Reef	Piscivorous	Evangelista et al.^14^
*Prionace glauca (PG)*	Blue shark	188.4 ± 12.1	65.5 ± 13.7	2	Pelagic	Oceanic	Piscivorous	This study
*Rhizoprionodon taylori (RT)*	Sharpnose shark	47.4 ± 12.0	0.6 ± 0.4	2	Pelagic	Coastal	Piscivorous	Evangelista et al.^14^
*Sphyrna zygaena (SZ)*	Smooth hammerhead	126.5 ± 8.5	7.7 ± 1.2	3	Pelagic	Coastal	Piscivorous	This study
*Trygonoptera imitata (TI)*	Eastern shovelnose stingaree	34.8 ± 0.8	1.3 ± 0.3	2	Benthic	Coastal	Non-piscivorous	Evangelista et al.^14^
*Urolophus paucimaculatus (UP)*	Sparsley-spotted stingaree	22.7 ± 0.9	0.4 ± 0.0	2	Benthic	Coastal	Non-piscivorous	Evangelista et al.^14^

Body mass and total length ± standard error, n-number, and the ecological categories (lifestyle, habitat, diet) assigned for the 26 species (abbreviation codes in parentheses) examined in this study.

### Morphological measurements

Magnetic resonance imaging (MRI) was used to examine inner ear morphology in situ. Following fixation, specimens were transferred to 0.1 M PB + 0.01% sodium azide for up to two weeks to remove excess fixative. Then, specimens were transferred to 0.1 M PB + 0.01% sodium azide with the addition of 5 mM of the contrast agent Prohance or Multihance (Bracco Diagnostics Inc., Princeton, N.J., USA) (from 1 week to up to 10 weeks, depending on the size of the sample, at 4 °C). Equilibrating the tissue in this contrast agent achieves a significant reduction in the longitudinal relaxation time (T1) of the sample and a corresponding increase in the SNR efficiency of the data acquisition in cartilaginous fishes (see^47,48^). MR image data was acquired from contrast-enhanced, fixed tissue.

Heads were removed from the contrast agent solution and scanned on either a Bruker Biospec 9.4 Tesla small animal scanner (using an 86 mm quad receiver coil) or a Siemens Magnetom 7 T whole body MR scanner (using a 32-channel head coil) at the Biomedical Research Imaging Center (BRIC) at the University of North Carolina Chapel Hill. For heads scanned on the 9.4 T, samples were embedded in an inert imaging media (Fomblin) prior to scanning. MR imaging consisted of a high-resolution (9.4 T: 70–90 μm; 7 T: 210–380 μm), T1-weighted anatomical acquisition, using a gradient recalled echo with no RF spoiling. The pulse sequence parameters used for this study are shown in Table S3.

**Figure 5:**
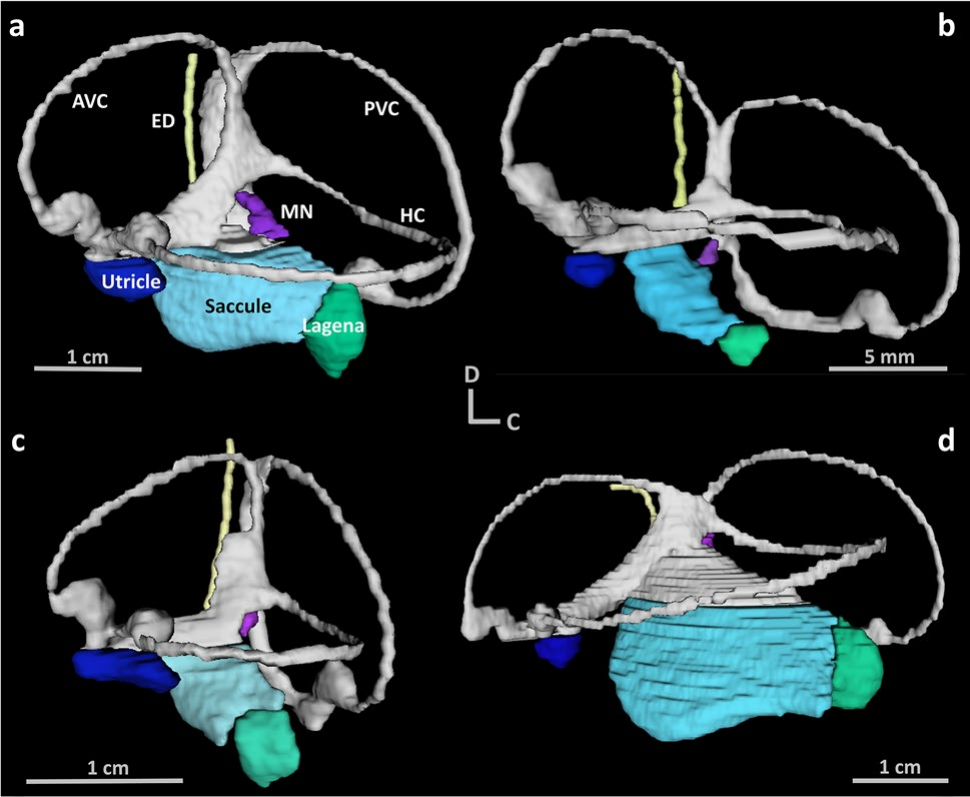
Three-dimensional visualisations.The inner ear from a (**a**) school shark (*Galeorhinus galeus*), (**b**) shortfin mako (*Isurus oxyrinchus*), (**c**) smooth hammerhead (*Sphyrna zygaena*), and (**d**) blue shark (*Prionace glauca*), illustrating the variation in different inner ear structures. AVC = anterior vertical canal, HC = horizontal canal, PVC = posterior vertical canal, MN = macula neglecta, ED = endolymphatic duct. All visualisations share the same orientation (D = dorsal, C = caudal).

Three-dimensional (3D) data acquired from MRI were digitally segmented using ITK-SNAP (Version 3.8.0), an interactive and open-source application that allows users to navigate 3D images and a toolbox for manual delineation and an interface for user-guided, semi-automatic segmentation using an active contour (level set) algorithm^128^. The inner ear end organs (saccule, lagena, utricle, macula neglecta), semi-circular canals (horizontal, anterior, and posterior), and endolymphatic duct were each segmented manually on consecutive scan slices across the three orthogonal planes to characterise the anatomy, acquire volumetric information of each labelled object, and create 3D visualisations of the inner ear (Fig. 5).

**Figure 6:**
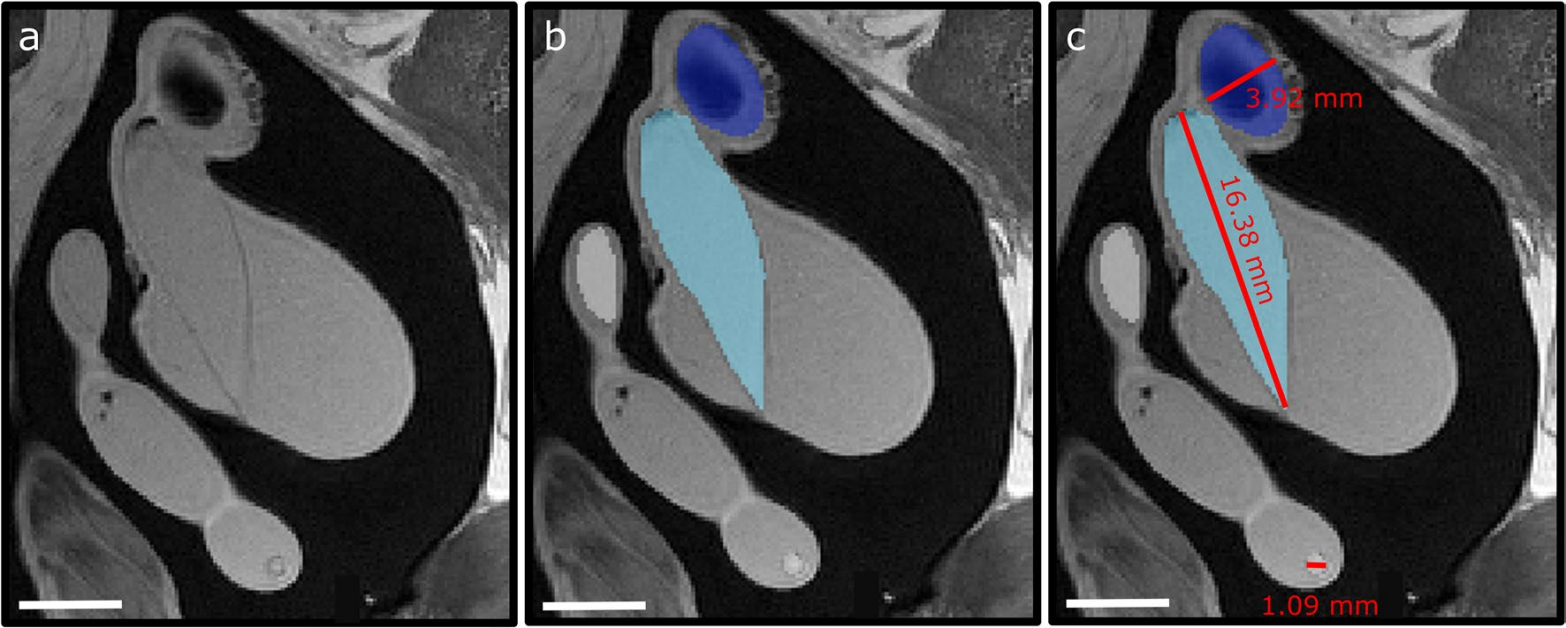
Linear measurement. An individual axial scan slice from the inner ear of a school shark (*Galeorhinus galeus*) showing (**a**) the original MR image, (**b**) the segmentation of inner ear structures, and (**c**) the linear measurement of the same inner ear structures. Dark blue = utricle; Light blue = saccule, Grey = semi-circular canals. Scale bar = 5 mm.

Using ITK-SNAP, linear measurements of inner ear structures were obtained to allow for direct comparisons with measurements from Evangelista et al.^14^ (Fig. 6). These linear measurements included the height and width of the otoconial organs, as well as the diameter of the semi-circular canals and their associated ampullae. The surface area of each end organ was then estimated by multiplying the length of the end organ by its respective width. While this method of estimation is coarse, actual surface area measurements (via whole mount imaging and ImageJ) and estimated surface area measurements (length × width via MRI) were independently assessed in individuals of two species, and similar results were obtained using both methods (t_14_ = 2.14, p = 0.744). Since the diameter of the three semi-circular canals and their ampullae were strongly correlated with each other (see Supplementary Data), diameter values from the three semi-circular canals and ampullae were averaged to obtain a single, mean canal diameter and a single mean ampullae diameter for each individual.

In addition, volumetric measurements (via MRI) were also obtained from the 10 newly examined species in this study. These measurements included volume of the saccule, lagena, utricle, macula neglecta, semi-circular canals, and endolymphatic duct (Fig. 5), as well the total volume of the inner ear (sum of all volumetric components) and the volume of the skeletal labyrinth (i.e., space within the otic capsule where the inner ear is located). A second data set was created, which contained only these volumetric measurements from 10 species. While this dataset was comprised of fewer species and thus held lower statistical power, it was useful in determining if general patterns observed in broader analyses of linear measurements (n = 26) were similarly identified in 3D measurements from a smaller number of species.

### Statistical analyses

In instances where data were available from more than one individual of the same species, data were averaged to obtain mean values for each species. Where possible, individuals of the same species were of similar body size and included both males and females, limiting the influence of intraspecific variation (*e.g.*,^61,63,65,66^). There were no significant differences in size-corrected surface area measurements (residuals) of otoconial organs between males and females (see Supplementary Data).

**Figure 7:**
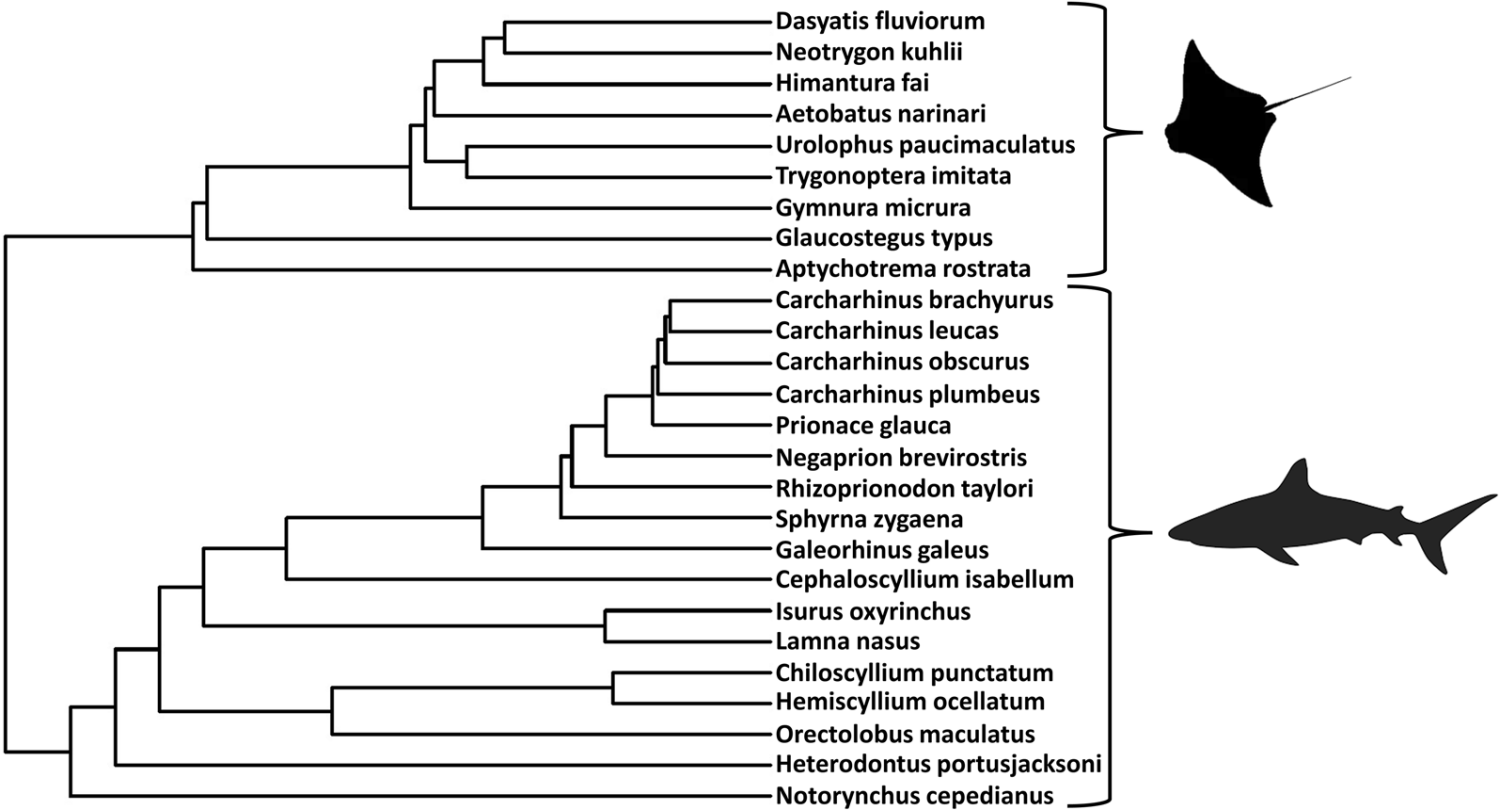
Phylogenetic tree used in this study. The 26 species of elasmobranchs examined in this study, created by pruning a larger (610 species) molecular tree (Stein et al.^132^) to the desired taxa set.

Species’ mean values were log_10_-transformed, as Akaike information criterion (AICc) scores (linear, log_10_-transformed, and square-root transformed) indicated log_10_-transformed data as the best fit^129,130^. As analyses without consideration of underlying phylogenetic relationships can result in overestimation of correlations and Type I errors^131^, log_10_-transformed data were assessed using phylogenetically informed approaches. A phylogeny of the 26 species examined in this study was created by pruning a comprehensive, molecular phylogeny of cartilaginous fishes from Stein et al.^132^. As *Cephaloscyllium isabellum* was not included in the larger tree, *C. isabellum* was assumed to be monophyletic with *C. ventrosium* (closely related species), and this branch was used (Fig. 7).

To determine if ecological factors influence inner ear morphology, each species was assigned to a primary habitat, lifestyle, and diet category, based on previously established criteria for cartilaginous fishes^30,37,69,133^ (see Table 3). Briefly, species were categorised by primary habitat (reef-associated, coastal, or oceanic), and lifestyle (benthic, or species that live solely in association with the seafloor; benthopelagic, or species that live on or near the seafloor, but also inhabit the water column; and pelagic, or species that live primarily in the water column). Diets were broadly separated into two categories: species that generally feed upon fishes (piscivorous) and species that feed mainly on invertebrates on the seafloor (non-piscivorous).

The scaling relationships between inner ear measurements and body size (mass) were assessed using a phylogenetic generalised least squares (pGLS) approach in the CAPER package in R^134^, with body size as the independent variable (measurement ~ body size). For each inner ear measurement, standardised phylogenetic residuals from the pGLS model were obtained to allow for size-independent comparison of traits between different ecological categories. Phylogenetic ANOVA was used to test for an influence of habitat, lifestyle, and diet on phylogenetic residuals, via the GEIGER package^135^. In addition, the phylogenetic dependence of residuals was assessed by determining Pagel’s lambda (λ) using the “phylosig” function from the phytools package^136^.

Variation in inner ear morphology was then assessed using a phylogenetic principal component analysis (pPCA) framework^133^. Absolute size of the inner ear and its various structures (surface areas of otoconial organs and mean canal and ampulla diameters) was analysed using pPCA in the phytools package in R, with total ear size as a covariate in subsequent analyses. We used the combined surface areas of the saccule, lagena, and utricle as a proxy for total ear size in surface area measurements, based on the full dataset (n = 26), while the summed volume of all inner ear structures represented total ear volume in the smaller subset of species (n = 10). The number of principal components that explained the majority (99%) of variation in inner ear morphology was then used to infer the major axes of inner ear organisation, as well as the relative importance of inner ear structures in explaining each axis. Phylogenetic PCA eigenvalues were also compared between habitats, lifestyles, and diets using phylogenetic ANOVA.

After exploring the influence of ecological explanatory variables on measured inner ear traits, the effect of habitat and diet on three axes was assessed on the full dataset (n = 26), and AICc scores were used identify the models that best explained the data^130^. Four candidate models of inner ear organisation were tested, which included structure ~ body mass + ear size (model 1), structure ~ body mass + ear size + diet (model 2), structure ~ body mass + ear size + habitat (model 3), and structure ~ body mass + ear size + diet + habitat (model 4). Model significance was determined using ΔAICc ≥ 2, with the lowest AICc score indicating the most supported model and differences within 2 units also considered as having substantial support^130^ (Table S7). Model-averaged coefficients were generated across the four candidate models for each of the three principal components using the MuMIn package in R^137^. Since ear size loaded heavily on the first principal component, explanatory models for PC1 contained only body mass as a covariate. After diet and habitat were identified as the most influential ecological traits, post hoc testing of estimated marginal means of individual factor levels for diet and habitat was performed using the emmeans package in R^138^. The slope effects of continuous variables (body mass and total ear size) and the intercept effects of categorical variables are presented relative to a reference level of coastal and piscivorous.

## Supplementary Information

Supplementary Information.

## Data Availability

All data needed to evaluate the conclusions in the paper are present in the main text and/or the Supplementary Materials. Detailed numerical data will be made available upon reasonable request of the corresponding author.
