# Supplementary material for: Quantitative assessment of inner ear variation in elasmobranchs
Source: Sci Rep. 2023 Jul 24;13:11939. doi: 10.1038/s41598-023-39151-0 (PMC10366120; doi:10.1038/s41598-023-39151-0)
Supplement: Supplementary file 1 — Supplementary Information. [file 41598_2023_39151_MOESM1_ESM.docx]

**Supplementary Text**

Males vs. females

T-tests indicated that there were no significant differences in size-corrected surface area measurements (residuals) of the otoconial organs between males and females (saccule surface area: t_55_ = 0.145, p = 0.885; lagena surface area: t_55_ = 0.411, p = 0.682; utricle surface area: t_57_ = -0.079, p = 0.937). See Table S9 for number of males and females of each species.

Canal and ampulla correlations

Regression of the diameter of the anterior semi-circular canal against the diameter of the posterior (F_1,24_ = 80.21, r^2^ = 0.77, p < 0.001) and horizontal semi-circular canal (F_1,24_ = 50.68, r^2^ = 0.68, p < 0.001) revealed significant positive relationships. In addition, regression of the diameter of the anterior canal ampulla against the diameter of the posterior (F_1,24_ = 185.73, r^2^ = 0.89, p < 0.001) and horizontal canal ampullae (F_1,24_ = 143.77, r^2^ = 0.86, p < 0.001) also revealed significant positive relationships.

**Table S1.** Scaling relationships between linear measurements and body mass across 26 elasmobranch species.

| **Trait** | **Slope** | **Intercept** | **DF** | **F-stat** | **R^2^** | **P-value** |
| --- | --- | --- | --- | --- | --- | --- |
| saccule surface area | 0.45 | -0.01 | 24 | 55.64 | 0.70 | <0.001 |
| lagena surface area | 0.49 | -0.93 | 24 | 116.5 | 0.83 | <0.001 |
| utricle surface area | 0.42 | -0.52 | 24 | 72.88 | 0.75 | <0.001 |
| total surface area | 0.45 | 0.16 | 24 | 30.69 | 0.56 | <0.001 |
| mean canal diameter | 0.11 | -0.57 | 24 | 14.77 | 0.38 | <0.001 |
| mean ampulla diameter | 0.15 | -0.16 | 24 | 24.74 | 0.51 | <0.001 |

**Table S2.** Scaling relationships between volume measurements and body mass across 10 shark species.

| **Trait** | **Slope** | **Intercept** | **DF** | **F-stat** | **R^2^** | **P-value** |
| --- | --- | --- | --- | --- | --- | --- |
| saccule volume | 0.91 | 1.50 | 8 | 30.88 | 0.79 | <0.001 |
| lagena volume | 0.79 | -1.96 | 8 | 73.92 | 0.9 | <0.001 |
| utricle volume | 0.74 | -1.69 | 8 | 59.26 | 0.88 | <0.001 |
| macula neglecta volume | 0.77 | -2.57 | 7 | 57.83 | 0.89 | <0.001 |
| horizontal canal volume | 0.70 | -0.39 | 7 | 51.45 | 0.88 | <0.001 |
| endolymphatic duct volume | 0.56 | -1.38 | 7 | 48.87 | 0.87 | <0.001 |
| total inner ear volume | 0.76 | -0.37 | 7 | 48.53 | 0.87 | <0.001 |
| skeletal labyrinth volume | 0.93 | -0.20 | 8 | 113.4 | 0.93 | <0.001 |

**Table S3.** Pulse sequence parameters for the T1-weighted scans of the specimens in this study, including echo time (TE), repetition time (TR), flip angle, number of averages (NEX), and isotropic resolution. For species where more than one specimen was scanned and parameters varied, a range is provided.

| **Species** | **Pulse Sequence** | **Scanner** | **n** | **TE (ms)** | **TR (ms)** | **Flip Angle (°)** | **NEX** | **Resolution (μm)** |
| --- | --- | --- | --- | --- | --- | --- | --- | --- |
| *Carcharhinus brachyurus* | FLASH 3D | 7T | 3 | 4.57-5.33 | 9.3-11 | 39-40 | 1-2 | 280-330 |
| *Cephaloscyllium isabellum* | FLASH 3D | 7T | 3 | 5.33 | 11 | 25-40 | 4 | 260 |
| *Galeorhinus galeus* | FLASH 3D | 7T | 3 | 5.33 | 11 | 36-48 | 2-4 | 320-330 |
| *Hemiscyllium ocellatum* | FLASH 3D | 9.4T | 3 | 4.3-7.2 | 95 | 76-80 | 4 | 75-90 |
| *Heterodontus portusjacksoni* | FLASH 3D | 9.4T | 3 | 7.3-8.8 | 60 | 63-66 | 3-5 | 70-90 |
| *Isurus oxyrinchus* | FLASH 3D | 7T | 3 | 5.33-5.41 | 11 | 40-48 | 4 | 210-300 |
| *Lamna nasus* | FLASH 3D | 7T | 1 | 5.33 | 11 | 40 | 4 | 290 |
| *Notorynchus cepedianus* | FLASH 3D | 7T | 1 | 5.33 | 11 | 34 | 4 | 370 |
| *Prionace glauca* | FLASH 3D | 7T | 2 | 5.33 | 11 | 40 | 4 | 380 |
| *Sphyrna zygaena* | FLASH 3D | 7T | 3 | 5.33 | 11 | 40 | 4 | 270-280 |

**Table S4.** Results from phylogenetic ANOVA, comparing relative linear measurements (residuals) of different inner ear structures between lifestyle, habitat, and diet categories across 26 elasmobranch species.

| **Trait** | **Factor** | **F-value** | **p-value** |
| --- | --- | --- | --- |
| saccule surface area | lifestyle | 3.62 | 0.263 |
| lagena surface area | lifestyle | 5.58 | 0.138 |
| utricle surface area | lifestyle | 2.50 | 0.413 |
| total surface area | lifestyle | 4.10 | 0.238 |
| mean canal diameter | lifestyle | 6.27 | 0.131 |
| mean ampulla diameter | lifestyle | 3.11 | 0.337 |
| saccule surface area | habitat | 0.91 | 0.547 |
| lagena surface area | habitat | 0.78 | 0.605 |
| **utricle surface area** | **habitat** | **4.54** | **0.043** |
| total surface area | habitat | 1.06 | 0.507 |
| mean canal diameter | habitat | 3.40 | 0.103 |
| mean ampulla diameter | habitat | 4.03 | 0.072 |
| saccule surface area | diet | 11.72 | 0.198 |
| lagena surface area | diet | 19.66 | 0.074 |
| utricle surface area | diet | 3.98 | 0.637 |
| total surface area | diet | 13.07 | 0.133 |
| mean canal diameter | diet | 0.345 | 0.806 |
| mean ampulla diameter | diet | 0.01 | 0.973 |

**Table S5.** Results from phylogenetic ANOVA, comparing relative volume (residuals) of different inner ear structures between lifestyle, habitat, and diet categories across 10 shark species.

| **Trait** | **Factor** | **F-value** | **p-value** |
| --- | --- | --- | --- |
| saccule volume | lifestyle | 0.92 | 0.534 |
| lagena volume | lifestyle | 1.17 | 0.456 |
| utricle volume | lifestyle | 0.43 | 0.736 |
| macula neglecta volume | lifestyle | 5.37 | 0.07 |
| **horizontal canal volume** | **lifestyle** | **6.77** | **0.029** |
| endolymphatic duct volume | lifestyle | 0.42 | 0.733 |
| total inner ear volume | lifestyle | 4.24 | 0.12 |
| skeletal labyrinth volume | lifestyle | 1.22 | 0.463 |
| saccule volume | habitat | 1.88 | 0.298 |
| lagena volume | habitat | 4.29 | 0.122 |
| **utricle volume** | **habitat** | **6.69** | **0.039** |
| macula neglecta volume | habitat | 3.58 | 0.113 |
| horizontal canal volume | habitat | 2.84 | 0.166 |
| endolymphatic duct volume | habitat | 0.20 | 0.872 |
| total inner ear volume | habitat | 2.09 | 0.279 |
| skeletal labyrinth volume | habitat | 1.90 | 0.338 |
| saccule volume | diet | 0.28 | 0.673 |
| lagena volume | diet | 0.00 | 0.991 |
| utricle volume | diet | 0.00 | 0.972 |
| macula neglecta volume | diet | 0.33 | 0.626 |
| horizontal canal volume | diet | 0.61 | 0.515 |
| endolymphatic duct volume | diet | 0.23 | 0.666 |
| total inner ear volume | diet | 0.54 | 0.568 |
| skeletal labyrinth volume | diet | 0.66 | 0.523 |

**Table S6.** Results from phylogenetic ANOVA, comparing pPCA eigenvalues for the three principal components between lifestyle, habitat, and diet categories across 26 elasmobranch species.

| **Trait** | **Factor** | **F-value** | **p-value** |
| --- | --- | --- | --- |
| PC1 eigenvalues | lifestyle | 6.21 | 0.112 |
| PC2 eigenvalues | lifestyle | 0.28 | 0.883 |
| PC3 eigenvalues | lifestyle | 4.59 | 0.203 |
| PC1 eigenvalues | habitat | 0.72 | 0.631 |
| PC2 eigenvalues | habitat | 0.09 | 0.935 |
| PC3 eigenvalues | habitat | 1.81 | 0.301 |
| **PC1 eigenvalues** | **diet** | **25.48** | **0.047** |
| PC2 eigenvalues | diet | 0 | 0.997 |
| PC3 eigenvalues | diet | 10.52 | 0.213 |

**Table S7.** Results of model selection for the three principal components.

| **Response** | **Candidate model variables** | **df** | **logLik** | **r2** | **AICc** | **delta** | **weight** | **lambda** |
| --- | --- | --- | --- | --- | --- | --- | --- | --- |
|  | **~body mass + diet + habitat** | **5** | **-8.08** | **0.90** | **29.20** | **0.00** | **0.83** | **0.00** |
| PC1 | ~body mass + habitat | 4 | -11.69 | 0.84 | 33.30 | 4.11 | 0.11 | 1.00 |
|  | ~body mass + diet | 3 | -14.10 | 0.81 | 35.30 | 6.12 | 0.04 | 0.97 |
|  | ~body mass | 2 | -15.98 | 0.78 | 36.50 | 7.31 | 0.02 | 0.98 |
|  | **~body mass + ear + diet + habitat** | **6** | **22.21** | **0.32** | **-28.00** | **0.00** | **0.61** | **0.00** |
| PC2 | ~body mass + ear | 3 | 16.33 | 0.54 | -25.60 | 2.44 | 0.18 | 0.00 |
|  | ~body mass + ear + habitat | 5 | 19.00 | 0.28 | -25.00 | 3.02 | 0.13 | 0.94 |
|  | ~body mass + ear + diet | 4 | 16.96 | 0.51 | -24.00 | 3.99 | 0.08 | 0.00 |
|  | **~body mass + ear** | **3** | **16.32** | **0.18** | **-25.50** | **0.00** | **0.44** | **0.54** |
| PC3 | **~body mass + ear + diet** | **4** | **17.54** | **0.02** | **-25.20** | **0.37** | **0.36** | **0.66** |
|  | ~body mass + ear + habitat | 5 | 18.22 | 0.13 | -23.40 | 2.11 | 0.15 | 0.43 |
|  | ~body mass + ear+ diet + habitat | 6 | 18.69 | 0.17 | -21.00 | 4.57 | 0.05 | 0.72 |

**Table S8.** Comparison of volume measurements of the soft tissue labyrinth (i.e., inner ear) with volume measurements of the skeletal labyrinth (i.e., space within the otic capsules).

| **Species** | **Soft labyrinth volume (mm^3^)** | **Skeletal labyrinth volume (mm^3^)** | **Ratio** |
| --- | --- | --- | --- |
| *Heterodontus portusjacksoni* | 14.43 | 55.26 | 0.26 |
| *Isurus oxyrinchus* | 148.04 | 2377.50 | 0.06 |
| *Hemicyllium ocellatum* | 3.77 | 10.33 | 0.36 |
| *Carcharhinus brachyurus* | - | 47722.80 | - |
| *Galeorhinus galeus* | 1004.67 | 5887.82 | 0.17 |
| *Cephaloscyllium isabellum* | 150.03 | 1082.13 | 0.14 |
| *Prionace glauca* | 2302.76 | 21196.45 | 0.11 |
| *Lamna nasus* | 101.47 | 937.18 | 0.11 |
| *Sphyrna zygaena* | 203.47 | 1203.97 | 0.17 |
| *Notorhynchus cepedianus* | 3562.62 | 28956.80 | 0.12 |

| **Species** | **n** | **Males** | **Females** | **Unknown** |
| --- | --- | --- | --- | --- |
| *Aetobatus narinari (AN)* | 2 | 0 | 1 | 1 |
| *Aptychotrema rostrata (AR)* | 4 | 2 | 2 | 0 |
| *Carcharhinus brachyurus (CB)* | 3 | 3 | 0 | 0 |
| *Carcharhinus leucas (CL)* | 1 | 0 | 1 | 0 |
| *Carcharhinus obscurus (CO)* | 1 | 0 | 1 | 0 |
| *Carcharhinus plumbeus (CPL)* | 3 | 0 | 2 | 1 |
| *Cephaloscyllium isabellum (CI)* | 3 | 0 | 3 | 0 |
| *Chiloscyllium punctatum (CP)* | 3 | 2 | 1 | 0 |
| *Dasyatis fluviorum (DF)* | 5 | 3 | 2 | 0 |
| *Galeorhinus galeus (GG)* | 3 | 1 | 0 | 2 |
| *Glaucostegus typus (GT)* | 3 | 0 | 3 | 0 |
| *Gymnura micrura (GM)* | 2 | 1 | 1 | 0 |
| *Hemiscyllium ocellatum (HO)* | 3 | 1 | 2 | 0 |
| *Heterodontus portusjacksoni (HP)* | 3 | 2 | 1 | 0 |
| *Himantura fai (HF)* | 2 | 2 | 0 | 0 |
| *Isurus oxyrinchus (IO)* | 3 | 1 | 2 | 0 |
| *Lamna nasus (LN)* | 1 | 0 | 1 | 0 |
| *Negaprion brevirostris (NB)* | 2 | 0 | 2 | 0 |
| *Neotrygon kuhlii (NK)* | 3 | 0 | 3 | 0 |
| *Notorynchus cepedianus (NC)* | 1 | 1 | 0 | 0 |
| *Orectolobus maculatus (OM)* | 5 | 1 | 4 | 0 |
| *Prionace glauca (PG)* | 2 | 2 | 0 | 0 |
| *Rhizoprionodon taylori (RT)* | 2 | 1 | 1 | 0 |
| *Sphyrna zygaena (SZ)* | 3 | 1 | 2 | 0 |
| *Trygonoptera imitata (TI)* | 2 | 1 | 1 | 0 |
| *Urolophus paucimaculatus (UP)* | 2 | 0 | 2 | 0 |

**Table S9.** Number of males and females for each species examined in this study.
